# HLA-DO as the Optimizer of Epitope Selection for MHC Class II Antigen Presentation

**DOI:** 10.1371/journal.pone.0071228

**Published:** 2013-08-08

**Authors:** Yuri O. Poluektov, AeRyon Kim, Isamu Z. Hartman, Scheherazade Sadegh-Nasseri

**Affiliations:** 1 Graduate Program in Immunology, Johns Hopkins School of Medicine, Baltimore, Maryland, United States of America; 2 Department of Pathology, Johns Hopkins School of Medicine, Baltimore, Maryland, United States of America; Oklahoma Medical Research Foundation, United States of America

## Abstract

Processing of antigens for presentation to helper T cells by MHC class II involves HLA-DM (DM) and HLA-DO (DO) accessory molecules. A mechanistic understanding of DO in this process has been missing. The leading model on its function proposes that DO inhibits the effects of DM. To directly study DO functions, we designed a recombinant soluble DO and expressed it in insect cells. The kinetics of binding and dissociation of several peptides to HLA-DR1 (DR1) molecules in the presence of DM and DO were measured. We found that DO reduced binding of DR1 to some peptides, and enhanced the binding of some other peptides to DR1. Interestingly, these enhancing and reducing effects were observed in the presence, or absence, of DM. We found that peptides that were negatively affected by DO were *DM-sensitive*, whereas peptides that were enhanced by DO were *DM-resistant*. The positive and negative effects of DO could only be measured on binding kinetics as peptide dissociation kinetics were not affected by DO. Using Surface Plasmon Resonance, we demonstrate direct binding of DO to a peptide-receptive, but not a closed conformation of DR1. We propose that DO imposes another layer of control on epitope selection during antigen processing.

## Introduction

Helper T cells recognize antigens as processed peptides bound to the groove of proteins of major histocompatibility complex class II (MHC II). MHC II αβ heterodimers form nonameric assemblies with Invariant chain (Ii) in the endoplasmic reticulum and are then transported through the Golgi complex to the endocytic pathway [1], [2]. During transport through the endocytic pathway the majority of Ii is removed from MHC II molecules by low pH and acid proteases [3] leaving a proteolytic fragment of Ii called CLIP (class II-associated Ii peptide) bound to MHC class II [4]. CLIP acts as a place-keeper for the MHC class II groove, inhibiting conformational changes that render the groove closed [5], [6], [7], [8], [9], [10], [11], [12], [13] and has to be removed in order to allow binding of exogenous peptides to nascent MHC class II complexes. Upon reaching the endosomal compartment the Ii chain is cleaved leaving behind only a small peptide fragment known as CLIP bound to the groove of the MHC molecule. DM, or H2-M in mice, is a non-classical HLA molecule and was discovered in B cell lines that were defective in Ag presentation by MHC class II molecules [14] and has been shown to play a critical role in the displacement of CLIP [15], [16], [17], [18]. In addition, DM transiently interacts with empty MHC class II to generate a peptide-receptive conformation, and plays an active role in the selection of specific peptide/MHC class II complexes during antigen processing [19], [20], [21], [22], [23], [24], [25], [26], [27], [28], [29], [30].

HLA-DO (H2-O in mice) (DO from now on) is another accessory molecule of the MHC Class II system. Like DM, DO is encoded in the highly polymorphic HLA region yet its sequence remains evolutionary conserved [31]. DO is expressed in B cells, the thymic epithelial cells and certain subsets of activated dendritic cells (DCs) [32], [33], [34]. The interest in DO was originally peaked when this molecule was discovered to be tightly associated with DM, raising thoughts about its function as a DM inhibitor. However, unlike DM knockouts, which established an important role for DM in promoting the exchange of CLIP for antigenic peptides and in thymic selection [16], [21] DO knockout mice have failed to demonstrate any clear immunological susceptibilities or advantages [35], [36], [37]. Hence the data on DO and its functions have remained controversial.

Much of the current knowledge on function of DO stems from studies with fibroblast cell lines transfected with MHC II related genes, including DO. A breakthrough was recently reported showing that when DO was overexpressed in dendritic cells of NOD mice; those mice gained an immunity to Type I diabetes, suggesting that DO expression eliminated presentation of self antigens that lead to diabetes [38]. These findings were consistent with many studies that have linked the effects of HLA-DO to the dampening of peptide binding to MHC II molecules through interaction with DM [39], [40]. Nonetheless, there are also some reports to the contrary, suggesting an enhancing peptide presentation for DO [35], [41], [42]. All studies on DO have assumed that DO functions through editing the activity of DM.

Here, using purified soluble MHC II HLA-DR1 (DR1), DO, and DM we determined the effects of DO individually, and in conjunction with DM on binding and dissociation kinetics of multiple peptides from DR1 molecules. Experiments testing the simultaneous effect of both DM and DO molecules on DR1/peptide complex formation suggests that DO interacts directly with DR molecules. We hypothesize that both DM and DO molecules work in concert to narrow down the peptide repertoire presented by MHC II molecules, with DO being the dominant editor having the final say over the selection process.

## Materials and Methods

### Production of Soluble MHC Class II Molecules

Soluble HLA-DR1was expressed in Hi5 insect cells through a Baculovirus expression vector and purified via L243 coupled resin as originally described [43]. For the purification of HLA-DM and HLA-DO, the protocol was modified to use the M2 FLAG (Sigma-Aldrich, St. Louis, MO, USA) and Ni-NTA (QIAGEN, Valencia, CA, USA) affinity columns respectively using gravity flow. Upon elution from the columns DM and DO molecules were concentrated (20–40 µM) in Citrate Phosphate pH 6.0 buffer with 0.05% NaN_3_ and stored frozen at −80°C in small aliquots. The soluble DR1 heterodimers were kept in PBS pH 7.4 with 0.05% NaN_3_.

### Peptides

Peptides used in our binding experiments at a minimum of 90% purity (Global Peptide Services, currently Pi Proteomics, Huntsville, AL, USA) were derived from characterized immunodominant HLA-DR1 (human MHC class II) binding peptides. The peptides were Fluorescein labeled at N-Termini as previously described [12]:

HA(306–318): CPKYVKQNTLKLAT (derived from Texas 77 flu strain HA protein peptide)

HA(Y308A): PKAVKQNTLKLAT

HA(anchorless): CPKAVKANGAKAAT

CII(259–273): CAGFKGEQGPKGEP (derived from Type II Collagen peptide used to induce arthritis in mice models)

H5N1-HA1(259–274): SNGNFIAPEYAYKIVK (derived from A/Vietman/1203/2004 flu strain peptide [44])

### Peptide Binding to DR1

Peptide binding experiments were performed as described previously [26], [28], [45], [46] based on efforts to simulate physiological conditions of antigen processing compartments whenever practically possible. In brief, we performed all binding experiments at 37°C in Citrate-Phosphate buffer, pH 5.0. Wt DR1 or mutant DR1, DR1βG86Y (1 µM) was incubated with fluorescent peptides (30 µM) in association experiments. For dissociation experiments we used DR1/fluorescent peptide complexes formed over 3 days incubation in 37°C, a competing non-fluoresceinated HA(306–318) peptide was added to the dissociation reaction at a concentration of 50 µM. In kinetics experiments where DO and DM were included, concentrations of 1 µM for DM and 4 µM DO or DM/DO were used. For each of the experimental combinations of DR1 with DM and DO, the binding of peptide to DR1 was measured at eight different time points: 10 h, 5 h, 3 h, 2 h, 1 h, 0.5 h, 0.25 h, 0 h, unless otherwise indicated. Excess unbound peptide was removed using Sephadex G-50 size-exclusion spin columns [26]. As a test for complete peptide removal as well as non-specific binding, all association assays included binding controls of peptide alone or with accessory molecules incubated for over 10 hours at 37°C. Binding control values were included in the corresponding figure legends. Fluorescence signal of peptide/DR1 complexes was measured by a Horiba FluoroMax®-3 instrument (Horiba Ltd., Kyoto, Japan) at 514–520 nm with an excitation wavelength of 492 nm and a 2 nm slit width.

### Surface Plasmon Resonance Experiments

SPR experiments were performed as described previously [46]. In brief, Anti-His tag (Invitrogen, Grand Island, NY, USA), Anti-FLAG tag (Sigma-Aldrich, St. Louis, MO, USA), and Anti-HA tag (Sigma-Aldrich, St. Louis, MO, USA) antibodies were immobilized on CM5 chips (GE Healthcare, Waukesha, WI, USA) through standard amine coupling procedures. Excess activated dextran carboxylate groups were capped with 2-amino-ethyl-sulfate (pH 8.0) to decrease the nonspecific binding of MHC molecules to the chip surface at a lower pH [47]. All protein solutions were diluted in the running buffer composed of Citrate-Phosphate pH 5.5 with 150 mM NaCl, 0.005% Tween-20 and 0.05% NaN_3_. Measurements were done at 27°C with flow rates ranging between 5–10 µL/min for all figures except 7 C and D, where the experiments were performed at 30°C and figure 8 where 37°C and 1 µL/min flow rates were used. MHC II proteins injected were all at 4 µM unless indicated otherwise.

## Results

### Soluble HLA-DO Molecules Expressed in Insect Cells form Stable Heterodimers Recognizable by a Conformational Specific Antibody

**Figure 1:**
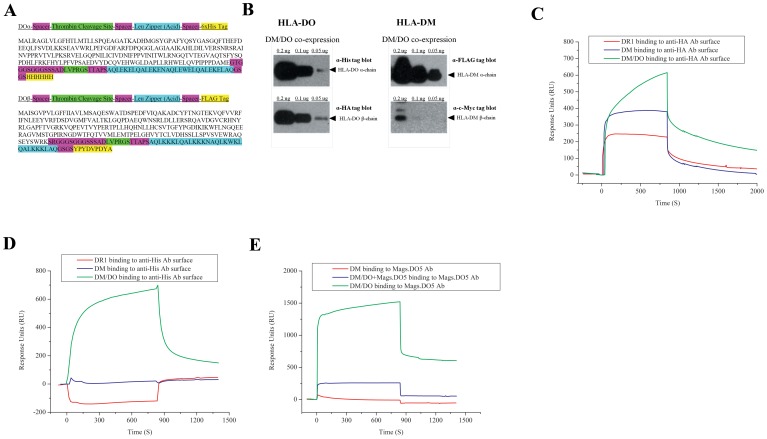
Recombinant soluble HLA-DO expression and characterization. (A) Design and sequence of the recombinant soluble HLA-DO α and β chain constructs. (B) Soluble co-expressed DO/DM complex was purified using Mags.DO5 monoclonal antibody affinity column. Four replicate samples were resolved on Bis-Tris SDS-PAGE gels in decreasing protein concentrations. Gels were blotted to PVDF membranes and stained for DO (left, anti-His and anti-HA) and DM (right, anti-FLAG and anti-c-Myc) specific tags. Data shown are representative of three independent experiments. (C) SPR sensograms of DR (red trace), DM (blue trace) and co-expressed DM/DO complexes (Mags.DO5 purified) (green trace) binding to anti-HA antibody coupled chip surfaces. (D) SPR sensgrams of binding of DR (red trace), DM (blue trace) and DM/DO (Mags.DO5 purified) (green trace) to anti-His antibody coupled chip surfaces. (E) SPR sensograms showing binding of DM/DO (Ni-NTA purified) (green trace), DM (red trace), and DM/DO (Ni-NTA purified) pre-bound to soluble Mags.DO5 (blue trace) to Mags.DO5 antibody coupled chip surfaces. The SPR experiments are representative of at least two independent trials.

It has been documented that DM is required for proper dimerization of the DO-alpha and DO-beta chains in addition to the transportation of the DO heterodimer from the ER to the specialized endosomal MHC class II compartments (MIICs) [48], [49]. To ensure dimerization of soluble DO molecules in the absence of DM, we incorporated leucine zipper acidic and basic chains into our DO constructs. The leucine zipper sequences were designed to link the DO alpha and beta chains coding sequences flanked by spacers one of which included a thrombin cleavage site. His- and HA- tags were added to the C-terminus of the DO alpha (MW ∼ 31 kDa) and beta (MW ∼ 32 kDa) chains respectively for the purpose of purification and identification (Fig. 1A). Soluble HLA-DO heterodimers were transiently expressed in Hi5 insect cells through a Bacculovirus vector and purified via its alpha chain His-tag by using the Ni-NTA affinity column through gravity flow. For different experiments, DO was expressed either alone or together with DM by simultaneously coinfecting Hi5 cells with viral vectors expressing each molecule. Interaction between soluble DO and DM was strong enough to allow for affinity purification of the DO/DM complexes on Ni-NTA resin. In addition, the conformational specific Mags.DO5 monoclonal antibody recognized the expressed DM/DO complexes. This antibody was originally generated upon immunizing mice with affinity-purified DM/DO complexes naturally expressed in B cells [50]. All four chains of DO and DM molecules were stained in western blots of DM/DO complex purified by Mags.DO5 (Fig. 1B).

Purified DM/DO complexes were further evaluated by binding to anti-HA and anti-His antibody immobilized chip surfaces using BIAcore Surface Plasmon Resonance (SPR) instrument (Fig. 1C and D). Another BIAcore SPR experiment demonstrated *real time* binding of DM/DO complexes to immobilized Mags.DO5 antibody surface (Fig. 1E). Injecting Ni-NTA column purified DM/DO complexes over Mags.DO5 mAb immobilized BIAcore chip surface resulted in approximately 600 Response Units (RU). To ensure that the observed binding was due to binding of DM/DO to Mags.DO5 mAb, we preincubated DM/DO complexes with soluble Mags.DO5 prior to injecting it over Mags.DO5 immobilized surface. As such, the RU signal generated by the binding of Mags.DO5 antibody to DM/DO complexes was reduced to about 100 RU, suggesting that the binding of DM/DO to Mags.DO5 immobilized surface was specific and that our insect-expressed recombinant DO folds with sufficient similarity to its B cell expressed counterpart to reproduce the natural epitope.

### Recombinant DO Tertiary Structure Displays the Necessary Epitope for Recognition of DM

**Figure 2:**
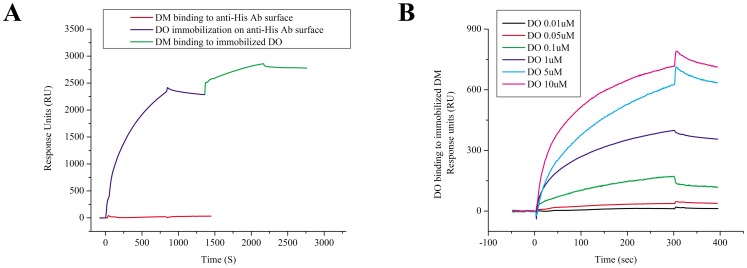
Soluble recombinant DO recognizes soluble recombinant DM. (A) DO (Ni-NTA purified) was immobilized on an anti-His antibody surface (blue trace). After a brief wash, DM was injected over the captured DO (green trace). A control injection of DM over anti-His antibody surface (red trace) showed no non-specific binding. Data shown are representative of six independent experiments. (B) DO (Ni-NTA purified) binding to DM immobilized by anti-FLAG antibody surface in concentrations ranging from 0.01 to 10 µM in separate experiments.

To discern whether purified soluble recombinant DO could bind to DM, DO was captured by anti-His tag antibody coupled SPR chip surface. After a brief 10 min run of wash buffer, soluble DM (4 µM) was injected over the DO surface, which resulted in 490 RU of DM binding (Fig. 2A). Then, the order of binding was reversed; DM was immobilized upon capture by an anti-FLAG tag antibody coupled chip surface, and DO was injected over the surface at 6 concentrations ranging from 0.01–10 µM (Fig. 2B). Once again, we observed formation of DM/DO complexes. It is of note that the binding of the DO solution (5 µM) to immobilized DM produced maximum binding of 500 RU, which was nearly identical to the binding of DM to immobilized DO. Similarities in binding of DM to DO and vice versa support the notion that the recombinant DO is structurally similar to its natural form.

### Effects of DO on Peptide Binding to DR1

**Figure 3:**
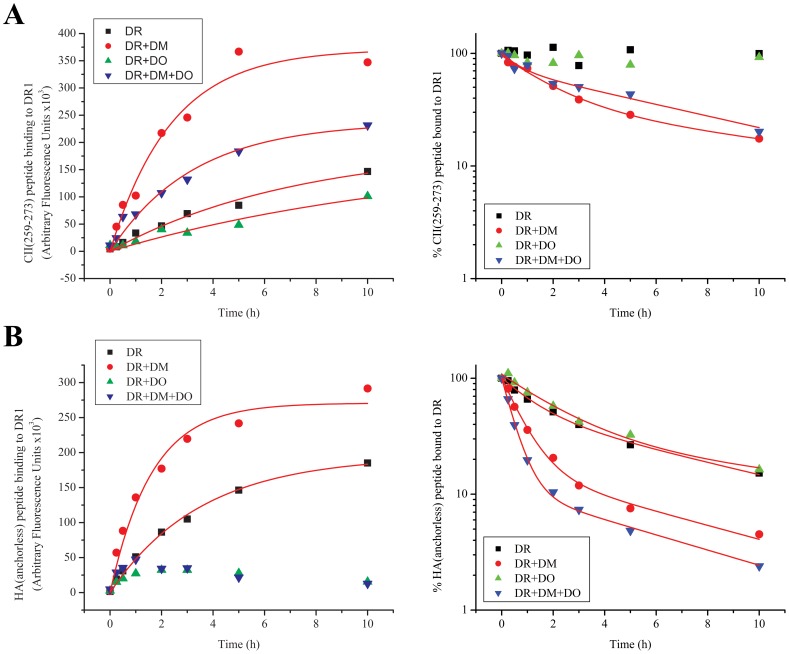
DO diminishes binding of peptides to DR1 molecules. (A) Association (left) and dissociation (right) of CII(259–273) peptide to DR1 molecules with no accessory molecules (black squares), with DM (red dots), with DO (green triangles), or both DO and DM (blue triangles) over the course of 10 hours. The fluorescence signals (Arbitrary Fluorescence Units) associated with the control samples incubated >10 hours in the absence of DR1 were measured: CII(259–273) peptide alone, 3996; CII(259–273)+DM, 1026; CII(259–273)+DO, 3326; CII(259–273)+DM+DO, 8278. (B) Association (left) and dissociation (right) of HA(anchorless) peptide to DR1 molecules with no accessory molecules (black squares), with DM (red dots), DO (green triangles) or both DO and DM (blue triangles) over the course of 10 hours. The fluorescence signals (Arbitrary Fluorescence Units) associated with the control samples incubated >10 hours in the absence of DR1 were measured: HA(anchorless) peptide alone, 3364; HA(anchorless)+DM, 1334; HA(anchorless)+DO, 1558; HA(anchorless)+DM+DO, 1726. Data shown are representative of at least three independent experiments.

We performed a number of peptide *association* and *dissociation* experiments with DR1 molecules to determine how DO would influence the binding kinetics of DR1 to various peptides. Fig. 3 depicts binding and dissociation of the immunodominant peptide of human Type II collagen CII(259–273) (Fig. 3A) [51], and a variant of influenza HA1, HA(306–318) peptide (HA(anchorless)). HA(anchorless) has all of its anchoring residues replaced with Alanine, pockets P1, P4, P7, and P9, or with Glycine, P6 (Fig. 3B) [43]. Control experiments for the detection of nonspecific binding by DO or DM were included and proved to be negligible. Addition of DO, or DM+DO had no effect on dissociation of either DR1/CII(259–273) or DR1/HA(anchorless) complexes. However, both DR1/peptide complexes showed ‘*DM-sensitivity*’, as defined by rapid dissociation in the presence of DM in the dissociation reactions (Fig. 3 right panels). The ability of DM to dissociate certain peptides from MHC II molecules while leaving others intact is directly related to the sequence of the peptide, specifically its P1 anchoring residue. It is possible to turn a peptide that is resistant to DM dissociation into a DM-*sensitive* one by simply replacing the P1 anchoring residue [26]. This allows for the classification of MHC II presented peptides by their DM sensitivity.

When DO was included in binding reactions, as expected, the maximum binding of both peptides to DR1 was diminished in comparison to no DO controls. DO diminished formation of DR1/peptide complexes during the 10 hours time-course in the presence, and the absence of DM for both peptides. This implies that the effect of DO can be quite significant and dominant over the effects of DM. In both cases DO diminished the ability of DR1 to bind and hence present peptides, as previously reported [35], [39], [52], [53].

**Figure 4:**
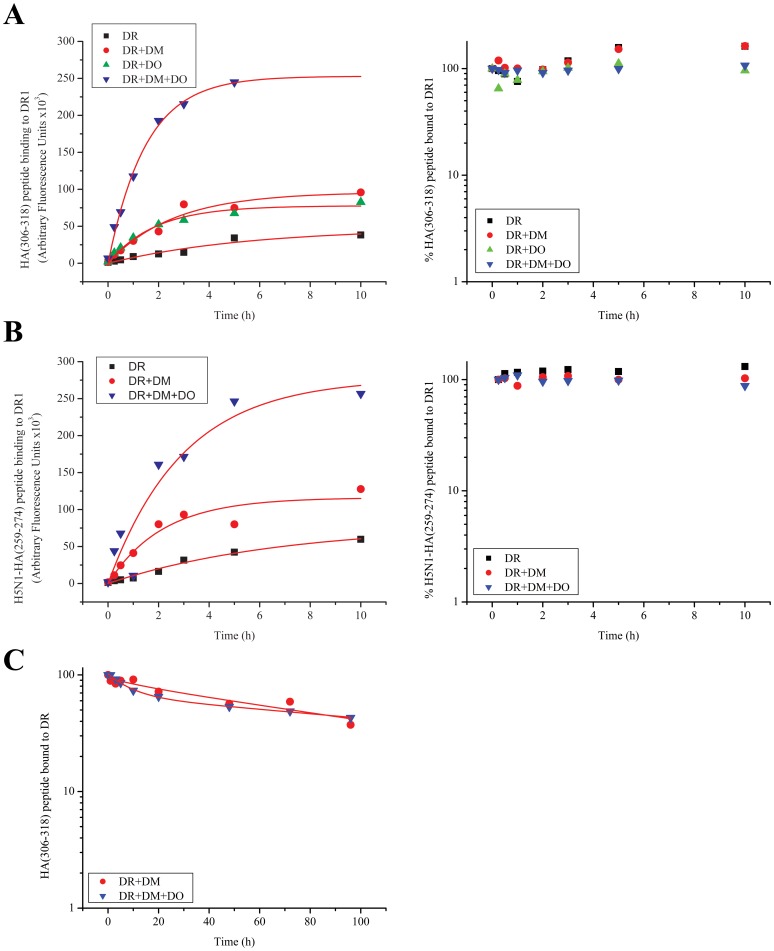
DO can increase the binding of peptides to DR1 molecules. (A) Association (left) and dissociation (right) of HA(306–318) peptide to DR1 molecules with no accessory molecules (black squares), with DM (red dots), DO (green triangles) or both DO and DM (blue triangles) over the course of 10 hours. The fluorescence signals (Arbitrary Fluorescence Units) associated with the control samples incubated >10 hours in the absence of DR1 were measured: HA(306–318) peptide alone, 1390; HA(306–318)+DM, 1376; HA(306–318)+DO, 3316; HA(306–318)+DM+DO, 9236. (B) Association (left) and dissociation (right) of H5N1-HA1(259–274) flu peptide to DR1 molecules with no accessory molecules (black squares), with DM (red dots), DO (green triangles) or both DO and DM (blue triangles) over the course of 10 hours. The fluorescence signals (Arbitrary Fluorescence Units) associated with the control samples incubated >10 hours in the absence of DR1 were measured: H5N1-HA1(259–274) peptide alone, 1312; H5N1-HA1(259–274)+DM, 1250; H5N1-HA1(259–274)+DM+DO, 9012. (C) Prolonged 96 hour dissociation experiment of HA(306–318) peptide from DR1 molecules with DM (red dots) or both DO and DM (blue triangles). Data shown are representative of at least three independent experiments.

We repeated the same experiment with two other peptides that form ‘*DM-resistant*’ complexes with DR1, i.e., DM does not affect their dissociation from DR1. HA(306–318) and H5N1-HA1(259–274)(SNGNFIAPEYAYKIVK), which has recently been identified and characterized [44] were examined in binding experiments. The results were opposite to the earlier experiment. DO affected peptide binding by increasing the binding maxima for both peptides leaving the association halftimes the same as in its absence. On the contrary, DO did not affect the dissociation of HA(306–318), or H5N1-HA(259–274) from DR1 during the 10 hour kinetic experiment (Fig. 4A and B), nor did it affect over the extended four days dissociation kinetic measurements (Fig. 4C), analogous to the previous two peptides that were *DM-sensitive*. In general all binding kinetics with or without DO displayed similar halftimes while the reactions reached different plateau levels. Lack of changes in binding half times in the presence of DO may suggest an increase in the fraction of DR molecules that become receptive in the presence of DO. The increase in complex formation cannot be attributed to a potential binding of peptides to DO because in control experiments in the absence of DR1, even after 10 hours of incubation of DO with peptides, the fluorescent signal was at the background levels (Fig. 4 legend). Surprisingly, the enhancement of peptide binding by DO was almost as large as the enhancing effect by DM. When both DM and DO molecules were included in the reaction the net effect was greater than with each molecule individually. The initial rates of the observed binding reactions were calculated from the slope of the linear fit of the of the early data points (∼ 1 hour) and were expressed as Arbitrary Fluorescence Units (AFU) per minute shown in a summary table (Table 1). It is noteworthy that although the rates vary significantly from one peptide to another, presence of DO enhanced the initial binding rates of HA(306–318) and H5N1-HA(259–274) peptides by 3–4 folds as compared to the initial rate of binding to DR1 alone.

**Table 1:** The initial rates of peptide/DR1 complex formation for tested peptides with and without accessory molecules.

Observed Initial Binding Rates in interaction of DR1 with Fluorescent Peptides#				
Peptides	DR1 alone	+ DM	+ DO	+ DM and DO
HA(306-318)	132	463	523	1779
HA(Y308A)*	118	331	184	234
HA(anchorless)	803	2141	385	651
H5N1-HA1(259-274)	98	665	257¶	2197
CII(281-295)	500	1593	137	1002

# Initial Rates were determined for 1 hour (Response; Arbitrary Fluorescence Units min^−1^).

* The effect of DO on HA(Y308A) was determined by using co-expressed DM/DO complex.

¶ Determined in a separate experiment.

### The Differential Effects of DO on Peptide Binding is Similar to the Effects of DM/DO

**Figure 5:**
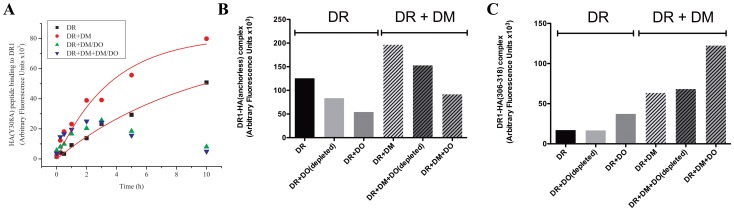
The observed effect of DO on peptide presentation is DO specific and can manifest in complex with DM. (A) Association of HA(Y308A) peptide to DR1 molecules in the presence of coinfected DM/DO complex (Ni-NTA purified). The peptide binding experiment was performed with no accessory molecules (black squares), with DM (red dots), DM/DO (green triangles), or both DM/DO and DM (blue triangles) over the course of 10 hours. The fluorescence signals (Arbitrary Fluorescence Units) associated with the control samples incubated >10 hours in the absence of DR1 were measured: HA(Y308A) peptide alone, 1140; HA(Y308A)+DM, 894; HA(Y308A)+DM/DO, 1404; HA(Y308A)+DM+DM/DO, 1944. (B) DO was depleted from a DO stock by immunoprecipitation via Ni-NTA followed by Mags.DO5 resin. The depleted sample was used instead of DO in reactions measuring HA(anchorless) peptide/DR complex formation in the presence or absence of DM after 5 hours of incubation. The fluorescence intensity of peptide/DR1 complexes formed in the DO depleted reaction was compared to a reaction containing no DO (left bar in each set of three), and a reaction that contained DO that did not undergo depletion (right bar in each set of three). The experiment is representative of three separate trials. (C) A DO-depleted sample was used instead of DO in a reaction measuring of HA(306–318) peptide/DR complex formation in the presence or absence of DM after 5 hours of incubation. The fluorescence intensity of peptide/DR1 complexes formed in the DO depleted reaction was compared to a reaction containing no DO (left bar in each set of three), and a reaction that contained DO that did not undergo depletion (right bar in each set of three). The experiment is representative of three separate trials.

A series of peptide association kinetics experiments were performed to determine if DO co-expressed with DM had similar effects on peptide binding compared to DO expressed in the absence of DM. DM/DO co-infection complexes were purified via Ni-NTA resin specific for the His-tag of DO-alpha. The peptide used in this experiment was HA(Y308A), which has a single substitution at P1 pocket and is sensitive to dissociation by DM [26]. Equimolar concentrations of DM/DO complexes, as with DO alone above, were added to the binding experiments. Additional free DM was added in the experiments with DM/DO and DM. DM/DO appeared to have the same effect on peptide loading as DO alone, in that it decreased the number of DR1 molecules able to bind HA(Y308A) (Fig. 5A). These results indicated that even in complex with DM, DO does not lose its function.

To ensure that the effects of DO observed in those experiments were because of DO and nothing else, we pre-depleted DO from our DO containing samples by tandem preincubations with Ni-NTA and Mags.DO5 antibody resins in tubes. After 20 minutes incubation with each resin, the supernatant fraction was collected and used to evaluate any effects it might have on peptide binding. The reducing and enhancing effects of the DO-depleted samples on peptide binding at the 5 hour time point (Fig. 5B and C, 2^nd^ and 5^th^ bars) were compared to control groups either excluding DO (Fig. 5B and C, 1^st^ and 4^th^ bars), or including DO samples that were not depleted of DO by immunoprecipitation (Fig. 5B and C, 3^rd^ and 6^th^ bars). It was confirmed that the removal of DO also removed the enhancing and diminishing effects of DO on DR1 binding to both HA(306–318) and HA(anchorless) peptides respectively. These results make it unlikely that the observed differential ability of DO to edit DR1 peptide binding is potentially due to any other molecules other than DO.

### HLA-DO Affects Peptide Binding by a Mechanism Different from DM

**Figure 6:**
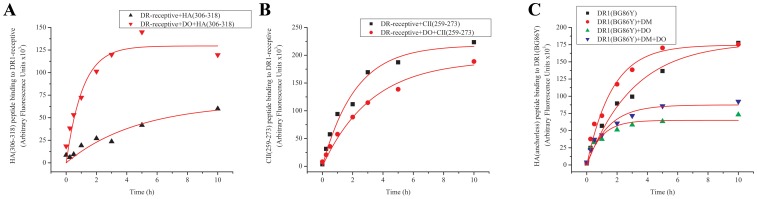
HLA-DO affects peptide binding to DR1 independent of DM. (A) Association kinetics of HA(306–318) to peptide-receptive DR1 without DO (black triangles) or with DO (red triangles). (B) Association kinetics of CII(259–273) to peptide-receptive DR1 without DO (black triangles) or with DO (red triangles). Experiments in A and B are representative of three-four trials. (C) HA(anchorless) association to constitutively receptive mutant DR1βG86Y molecules with no accessory molecules (black squares), with DM (red dots), DO (green triangles) or both DO and DM (blue triangles). The fluorescence signals (Arbitrary Fluorescence Units) associated with the control samples incubated >10 hours in the absence of DR1 were measured: HA(anchorless) peptide alone, 1804; HA(anchorless)+DM, 1512; HA(anchorless)+DO, 2280; HA(anchorless)+DM+DO, 2888.

The above experiments have provided evidence that DO works directly on DR1 and not by modulating the effect of DM as previously proposed [35], [39], [52], [53]. However, since DO does not enhance the dissociation of DR/peptide complexes, we propose that DO may work on DR molecules that have been altered by DM to adopt a ‘*peptide-receptive*’ conformation [12], [54]. To test this hypothesis, we used DR1 in a ‘*peptide-receptive*’ conformation (DR-receptive), which was generated in the absence of DM. DR1 molecules were incubated with unlabeled HA(anchorless) peptide for three days, isolated and used in peptide association experiments. Because of the rapid dissociation of HA(anchorless)/DR1 complexes (T1/2 ∼90 min), a significant number of DR1 molecules convert to a DR-receptive state within a short time [12]. The results shown in Fig. 6A and 6B demonstrate that indeed a DR-receptive conformation acts as a good substrate for DO. DO enhanced binding of HA(306–318) peptide to DR-receptive molecules, while in parallel experiments, DO reduced the binding of CII(259–273) peptide to the receptive conformer. These effects were comparable to the effects of DO in the presence of DM as shown in Fig. 4A and Fig. 3A.

To address how DO interacts with peptide-receptive DR, and whether DO could alter the receptive state, we took advantage of the mutant DR1βG86Y molecule that has a filled P1 pocket and is constitutively in a receptive conformation [26], [43], [45]. DR1βG86Y molecule binds peptides following a single exponential kinetic model similar to the peptide-receptive wild-type DR1 conformer, or DR molecules in the presence of DM. Because of its shallow P1 pocket, only peptides that have small corresponding side chains may bind to DR1βG86Y, and because of having a rigid P1, DR1βG86Y molecule is resistant to the effects of DM in peptide association and dissociation. Fig. 6C demonstrates that DO reduces the binding of HA(anchorless) to DR1βG86Y as it does for the wild type DR1. Because the mutant DR1 molecule does not close, these observations suggest that DO inhibits the binding of HA(anchorless) to DR1 through a mechanism that does not involve closing of the peptide binding groove.

### HLA-DO Binds to DR-receptive but not to DR-closed Molecules

**Figure 7:**
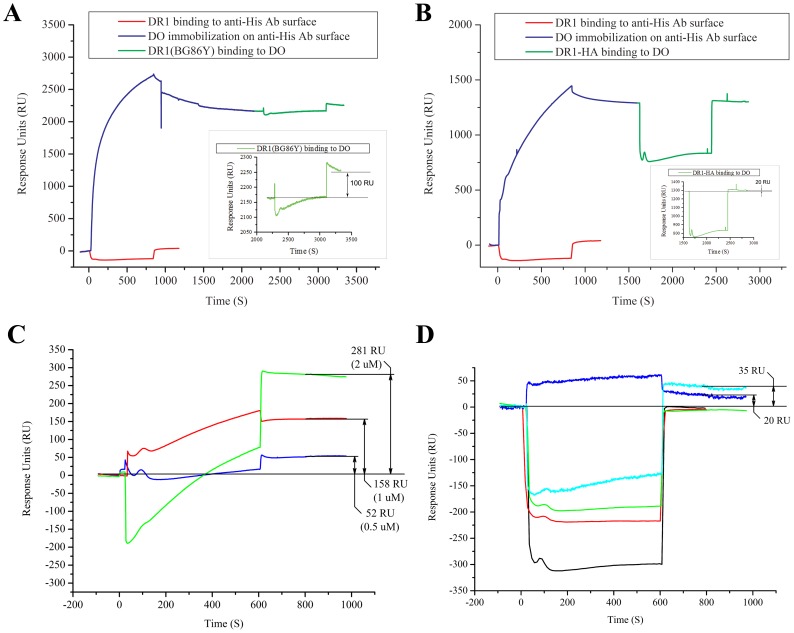
HLA-DO interacts with DR1 in a receptive conformation but not a peptide-loaded, compact form. (A) SPR sensograms of constitutively receptive DR1βG86Y (4µM) binding to DO. Ni-NTA purified DO was immobilized on anti-His antibody coupled chip (blue trace). After a brief wash, DR1βG86Y was injected over the captured DO surface (green trace). An injection of unloaded DR1 over the anti-His antibody surface (red trace) was performed to control for potential nonspecific binding DR1 to the chip surface. (B) SPR sensograms of closed compact DR1/HA(306–318) complex (4µM) binding to DO. Ni-NTA purified DO was immobilized on anti-His antibody coupled chip (blue trace). After a brief wash, DR1/HA(306–318) was injected over the captured DO surface (green trace). An injection of unloaded DR1 over the anti-His antibody surface (red trace) was performed to control for potential nonspecific binding DR1 to the chip surface. (C) DR1βG86Y binding to DM/DO complex molecules. Mags.DO5 purified DM/DO was immobilized on anti-His antibody coupled chip surface to a level of 2000–3000 RU. After a brief wash, DR1βG86Y was injected over the captured DM/DO at concentrations of 0.5 µM (blue trace), 1 µM (red trace), 2 µM (green trace). Before every injection of DR1βG86Y, the DM/DO molecules captured on the surface were regenerated to insure that the surface was not saturated by bound DR1 molecules. The signal of the resulting binding ∼200–300 after the end of the injection is marked on the graph. (D) Binding controls of DR1βG86Y and DR1/HA(306–318) with anti-His antibody, DM/DO and DM surfaces. Following the immobilization of anti-His antibody, 4 µM DR1βG86Y (red trace) and 4 µM DR1/HA(306–318) (black trace) were injected over the immobilized antibody. Upon capturing 2000–3000 RU of DM/DO by the anti-His antibody, 4 µM DR1/HA(306–318) was injected over the DM/DO (green trace). In a separate control, 3000 RU of DM was captured by immobilized anti-FLAG antibody. 4 µM DR1βG86Y (cyan trace), or 4 µM DR1/HA(306–318) (blue trace) was injected over the captured DM. The magnitude of binding was measured at the *stability point* ∼200–300 seconds after the end of the injection. Data shown are representative of two independent experiments.

Our data suggest a mechanism where DO would interact with a receptive DR1 molecule but not with its closed peptide bound conformation. To test this notion directly, we performed an SPR experiment to examine any binding interactions between DO and DR1 in either a closed or a receptive state in *real time*. DO was captured by anti-His antibody coupled surface. DR1βG86Y molecules (DR-receptive conformer) was then injected over the surface, which yielded 100 RU binding (Fig. 7A). However, when DR1 in complex with HA(306–318) peptide (DR-closed conformer) was injected over the DO bound surface the binding was merely 20 RU (Fig. 7B), which is within background levels observed in our negative controls, i.e., DR1 (no His tags) binding to anti His surface.

In a separate SPR experiment, the direct interaction of DR1βG86Y molecules with DM/DO was measured. DM/DO molecules were captured by immobilized anti-His antibody surface to a total signal of 2000–3000 RUs. Then, DR1βG86Y molecules were injected in three concentrations of 0.5, 1 and 2 µM over the DM/DO surface (Fig. 7C). Repeat injections of increasing concentrations of DR1βG86Y were performed on the same flow cell following the prolonged dissociation of the bound DR molecules (0.5 µM) or a regeneration of the surface with the injection of pH 11.5 CAPS buffer, washing the surface overnight in running buffer and recapture of 2000–3000 RUs of DM/DO molecules before the next injection (1 µM). With increasing concentration of DR1βG86Y more binding was measured at the *stability point*, i.e., the point where protein injection stopped and running buffer started.

Binding controls were included to account for any possible non-specific binding that may have occurred. Neither DR1βG86Y at 4 µM when injected over anti-His antibody coupled surface, nor 4 µM of pre-formed DR1-HA complexes (closed conformer) injected over the DM/DO bound surface resulted in noticeable binding. Finally, to make sure that the observed binding was specific to DO and not DM in complex with DO, DR1βG86Y and DR1-HA molecules were injected over DM molecules captured by a anti-FLAG antibody coupled chip surface (Fig. 7D). The binding of DR1-HA molecules to DM remained at a minimal level of 20 RU, whereas DR1βG86Y binding to DM resulted in 35 RU. In each case the signals were significantly smaller than those resulting from DR1(βG86Y) binding to DM/DO complexes at lower concentrations.

**Figure 8:**
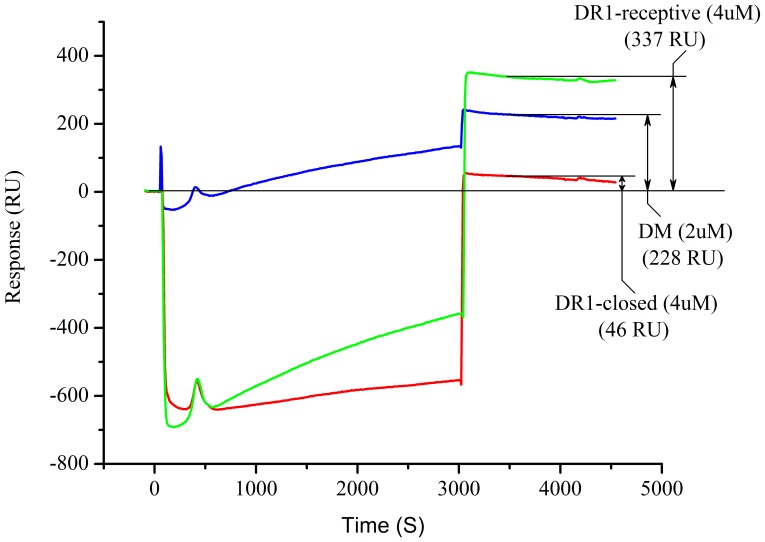
Transiently receptive DR1 molecules interact with DM/DO complexes. Mags.DO5 purified DM/DO was immobilized on anti-His antibody coupled chip surface to a level of 2000–3000 RU. Receptive DR1 molecules were generated from DR1/HA(Y308A) peptide complexes by removing the excess peptide by G-50 size-exclusion spin columns, adding DM and allowing the peptide to dissociate for 20 minutes at 37°C. Immediately after the 20 minute incubation 4 µM receptive DR1 with 2 µM DM was injected over the captured DM/DO at a rate of 1 µL/min and a constant flow-cell temperature of 37°C for 50 minutes (green trace). As a control, prior to the injection of receptive DR1 molecules, 2 µM DM (blue trace), or 4 µM DR1/HA(306–318) (red trace) were injected at the same flow rate and temperature. Data shown are representative of two independent experiments.

Alternatively, an even more sensitive experiment was performed to validate that DO molecules or DM/DO complexes bind DR1 in a receptive but not a closed conformation. For this purpose a transient receptive conformation was generated in DR1 molecules by pre-loading them with DM-sensitive HA(Y308A) peptide for 3 days at 37°C (DR1/HA(Y308A)). Before injection into the BIAcore instrument, excess HA(Y308A) peptide was removed through G50 column filtration. DM (2µM) was then added to the DR1(4 µM)/HA(Y308A) complexes as they were incubated at 37°C for 20 minutes. Due to the short half-time of dissociation of HA-Y308A and the addition of DM, most of the peptide would dissociate from DR1 molecules leaving them in an open peptide-receptive conformation. Immediately after the 20 minute incubation this solution, which contained DR1-receptive molecules, DM, and the unbound HA(Y308A) peptide was injected over DM/DO captured surface. By slowly injecting DR1 molecules at a rate of 1 µL/min for 50 minutes we saw a 337 RU binding of DR1-receptive to DM/DO. Since the receptive conformation is transient and may not be adopted by all the DR1 molecules, unlike with DR1βG86Y, the injection had to be continued over a prolonged period of time while keeping the temperature of the flow-cell at 37°C to allow further generation of receptive DR1 during the injection (Fig. 8). Control injections of 4 µM DR/HA closed conformer under the same conditions produced only 46 RU binding. Because DR-receptive molecules included DM, we also injected DM (2 µM) as control, which resulted in 228 RU of binding. We believe this rather high level of DM binding to DM/DO surface is due to dissociation of DM from the anti-His captured DM/DO complexes over long incubation at 37°C [57].

Of note, even while in complex with DM, DO does not lose its ability to interact with receptive DR1βG86Y molecules. Consistent with our hypothesis, these results suggest that DO exclusively interacts with the DR1 conformers in a receptive state.

## Discussion

DO, an accessory molecule in MHC class II antigen presentation pathway, has long been described as an inhibitor of DM. Our findings provide evidence to the contrary, demonstrating that DO interacts with DR molecules directly, and not through modulating the effect of DM. Further, we show that DO can have both enhancing and diminishing effects on the formation of peptide-DR complexes based on the structure of the peptide. Interestingly, within the scope of our experiments, peptides augmented by DO were *DM resistant* and those whose bindings were reduced by DO were *DM sensitive*.

Through multiple strategies, we provided evidence that our recombinant DO is properly folded: First, the co-expressed DO/DM complex purified via DOα- was fully recognized by conformational specific Mags.DO5 antibody. Second, our singly expressed DO bound to DM in *real time* SPR experiments, an indication of its correct folding and biological relevance. Third, we also have ruled out the possibility that our measured peptide binding occurs due to molecules other then DR. We find it unlikely that the enhanced binding of DM-resistant peptides to DR1 might be due to nonspecific peptide binding to a region in the DO-DR interface [55]. If this were the case, we would not have been able to see a diminishing effect of DO on DM-sensitive peptides. Our DO depletion studies showed that the observed differential ability of DO in editing DR1 peptide binding is not due to any other molecules besides DO itself.

Current models propose an inhibitory role for DO through inhibition of DM functions [56], [57], [58]. We have dissected the regulatory roles of DO on *association* and *dissociation* of peptides and we demonstrate that DO, unlike DM, affects only binding, and not the dissociation of peptide/DR complexes. It is unlikely that DO would inhibit DM while at the same time having no effects on the dissociation of peptides from DR molecules. More importantly, we find that DO has the same enhancing and diminishing effects on peptide binding in the absence of DM. These results, for the first time, provide a mechanistic insight into how DO modulates antigen presentation; rather than being a simple inhibitor of DM by sequestration into DO/DM complexes, we show that DO is fully active even while in complex with DM.

Here, we propose a molecular mechanism for DO-assisted peptide presentation in an MHC Class II system. DO has a positive effect on the binding of HA(306–318) peptide, whereas its effect becomes negative when the P1 pocket residue is replaced by Ala as in the case of HA(Y308A) and HA(anchorless) peptides. Given how important the role of P1 pocket is for its sensitivity to DM [26], [28], [30], [59], [60], [61], one may speculate that the function of DO is intertwined with that of DM. As established by numerous studies, the major role of DM in peptide binding is to enhance dissociation of certain peptides and to generate peptide-receptive conformers of MHC II. Hence, DO and DM collaborate to optimize epitope selection as can be seen by the increased peptide binding that occurs when both DM and DO present. This function of DO as a secondary peptide repertoire editor, lends itself to a better explanation of why the genes of DO and DM have been evolutionary conserved [31], [62].

**Figure 9:**
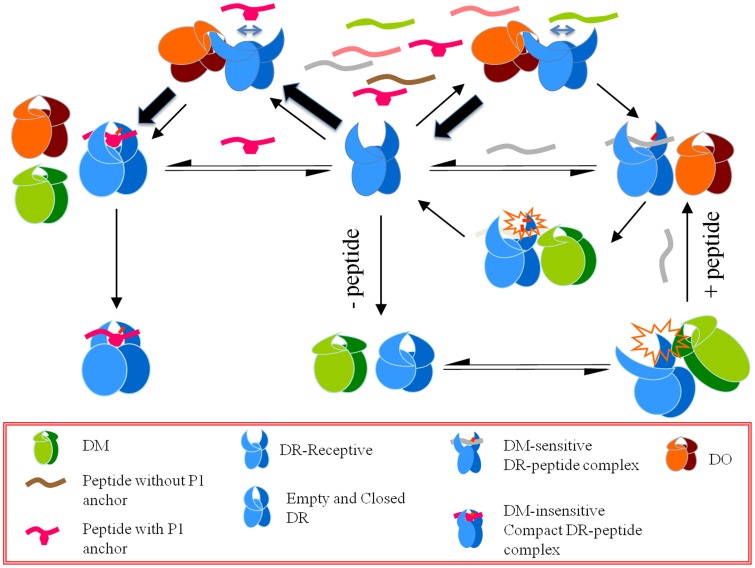
A model for the effects of HLA-DO on antigen presentation. DO likely interacts with peptide-receptive DR molecules and may stabilize an overly receptive conformation. This conformation lends itself to a more efficient release of the poorly binding peptides while helping the formation of compact complexes with the well-fitting peptides. In the pool of available peptides those with weak anchoring residues that tend to be more DM-sensitive may not get a chance to stabilize in the groove, and therefore are outcompeted by DM-resistant peptides with bulkier hydrophobic P1 pocket residues. We theorize that DO interacts primarily with DR molecules in receptive conformation mostly generated by the effector function of DM.

Our proposal regarding cooperativity between DO and DM is supported by the observation that DO diminishes the binding of HA(anchorless) to the mutant DR1βG86Y molecules despite its constitutively open groove. Additionally, SPR experiments detected the binding of DO to receptive, but not to closed/compact peptide/DR1 complexes in a dose-dependent manner. We have demonstrated by two different approaches that once DR1 is in receptive conformation (DR-receptive or DR1βG86Y) it binds to DO either alone, or in complex with DM. We hypothesize that the function of DO is greatly enhanced by the effects of DM, which in addition to its own peptide repertoire editing activities serves as the inducer of ‘DR-receptive’ conformation. Hence, we suggest that DO interacts with DR groove in peptide-receptive conformation and stabilizes yet another intermediate conformation. This new intermediate conformation lends itself to a less efficient placement of poorly binding peptides within the groove. Among our pool of tested peptides those with suboptimal P1 pocket residues (DM-sensitive), may not get a chance to stabilize themselves in the groove. As such, they are outcompeted by DM-resistant peptides bearing optimal P1 pocket residues. Based on these hypotheses we are presenting a model for the mechanism of DO function (Fig. 9).

Reports of in vivo results support our proposed mechanism: DO expression in B cells was reported to have both enhancing and diminishing effects on B cell entry into the germinal centers depending on two different peptides recognized by follicular helper T cells [63]. In addition, variable modulating effects of DO on peptide selection [41], [64], as well as the lack of detectable changes in CLIP/MHCII expression in DO^−/−^ B cells point to the positive and negative roles of DO in antigen presentation [35], [36], [42]. A recent elegant study reported a co-crystal for DM/DO complexes and provided insights into the DM and DO interface [55]. The same study suggested that DO inhibits the function of DM by serving as a competitive inhibitor for DM/DR interactions although, authors did not provide experiments that would examine the effects of DO in the absence of DM.

In conclusion, we have documented a novel function for DO in epitope editing, and have presented a working model for the cooperative interactions of DO and DM. Based on our model, the antigenic selection imposed by DO takes precedence over the selection favored by DM, empowering DO with the “final cut” over the selection of peptides to be displayed on the antigen presenting cells. We speculate that having two accessory molecules to edit the MHC Class II peptide repertoire would narrow the number of unique peptides presented to T cells. A restricted response to a given antigen might be necessary for keeping the sheer number of long-lived memory T cells specific to an antigen small enough to be accommodated within the limited space of lymph nodes [65]. The expression of DO in B cells could easily be explained by the need to limit the number of long-lived memory T cells developed against different antigens [66], [67], [68]. Perhaps DO expression in the thymus medulla would ensure efficient negative selection in the thymus against T cells specific to self-antigens [69]. While the greater function of DO in our immune system requires further exploration, in certain cases this effect could be significant enough to stop the onset of an autoimmune disease [38] or exacerbate it [70]. DO is a new lever that could help to control the antigenic repertoire generated by the immune system.
